# Incidence of cardiometabolic outcomes among people living with HIV‐1 initiated on integrase strand transfer inhibitor versus non‐integrase strand transfer inhibitor antiretroviral therapies: a retrospective analysis of insurance claims in the United States

**DOI:** 10.1002/jia2.26123

**Published:** 2023-06-12

**Authors:** Peter F. Rebeiro, Bruno Emond, Carmine Rossi, Brahim K. Bookhart, Aditi Shah, Gabrielle Caron‐Lapointe, Marie‐Hélène Lafeuille, Prina Donga

**Affiliations:** ^1^ Divisions of Infectious Diseases & Epidemiology Department of Medicine Department of Biostatistics Vanderbilt University Nashville Tennessee USA; ^2^ Analysis Group, Inc. Montréal Québec Canada; ^3^ Janssen Scientific Affairs LLC Titusville New Jersey USA

**Keywords:** treatment, ARV, cohort study, North America, cardiovascular diseases, metabolic disease

## Abstract

**Introduction:**

Integrase strand transfer inhibitor (INSTI)‐containing antiretroviral therapy (ART) has been associated with weight gain, though there is limited information on associations between ART‐related weight gain and cardiometabolic outcomes among people living with HIV‐1 (PLWH). We, therefore, evaluated risks of incident cardiometabolic outcomes following INSTI versus non‐INSTI‐based ART initiation in the United States.

**Methods:**

We conducted a retrospective study using IBM MarketScan Research Databases (12 August 2012−31 January 2021). Treatment‐naïve PLWH initiating ART (index date) on/after 12 August 2013 (approval date of the first second‐generation INSTI, dolutegravir) were included and censored at regimen switch/discontinuation, end of insurance eligibility or end of data availability. We used inverse probability of treatment weights constructed with baseline (12 months pre‐index) characteristics to account for differences between INSTI‐ and non‐INSTI‐initiating cohorts. Doubly robust hazard ratios (HRs) obtained from weighted multivariable Cox regression were used to compare time to incident cardiometabolic outcomes (congestive heart failure [CHF], coronary artery disease, myocardial infarction, stroke/transient ischemic attack, hypertension, type II diabetes, lipid disorders, lipodystrophy and metabolic syndrome) by INSTI‐initiation status.

**Results:**

Weighted INSTI (mean age = 39 years, 23% female, 70% commercially insured, 30% Medicaid insured) and non‐INSTI (mean age = 39 years, 24% female, 71% commercially insured, 29% Medicaid insured) cohorts included 7059 and 7017 PLWH, respectively. The most common INSTI‐containing regimens were elvitegravir‐based (43.4%), dolutegravir‐based (33.3%) and bictegravir‐based (18.4%); the most common non‐INSTI‐containing regimens were darunavir‐based (31.5%), rilpivirine‐based (30.4%) and efavirenz‐based (28.3%). Mean±standard deviation follow‐up periods were 1.5±1.5 and 1.1±1.2 years in INSTI‐ and non‐INSTI‐initiating cohorts, respectively. INSTI initiators were at a clinically and significantly increased risk of experiencing incident CHF (HR = 2.12, 95% confidence interval [CI] = 1.08−4.05; *p* = 0.036), myocardial infarction (HR = 1.79, 95% CI = 1.03−5.65; *p* = 0.036) and lipid disorders (HR = 1.26, 95% CI = 1.04−1.58; *p* = 0.020); there was no evidence of an increased risk for other individual or composite outcomes.

**Conclusions:**

Over a short average follow‐up period of <2 years, INSTI use among treatment‐naïve PLWH was associated with an increased risk of several cardiometabolic outcomes, such as CHF, myocardial infarction and lipid disorders, compared to non‐INSTI use. Further research accounting for additional potential confounders and with longer follow‐up is warranted to more accurately and precisely quantify the impact of INSTI‐containing ART on long‐term cardiometabolic outcomes.

## 1 INTRODUCTION

In the United States (US), there are approximately 1.2 million people living with HIV‐1 (PLWH) [1]. Antiretroviral therapy (ART), including protease inhibitors (PIs), non‐nucleoside reverse transcriptase inhibitors (NNRTIs) and integrase strand transfer inhibitors (INSTIs) [2] have been shown to decrease the risk of HIV‐1 transmission, improve clinical outcomes and contribute to a better quality of life among PLWH [3, 4, 5, 6]. The 2022 US Department of Health and Human Services (DHHS) guidelines for the use of antiretrovirals recommend one of two INSTIs as part of a complete ART regimen for most ART‐naïve PLWH: bictegravir or dolutegravir, although for those with HIV RNA >500,000 copies/ml, dolutegravir/lamivudine is not recommended [7]. In specific clinical situations (i.e. needing to start treatment prior to drug resistance testing or in situations where concerns about treatment adherence exist), regimens containing darunavir (a PI) are also recommended.

Despite the recommendation for first‐line use in most PLWH, INSTI‐based regimens are associated with a number of clinical events, including weight gain [7, 8, 9, 10]. In a US study using claims and electronic medical records, ART‐naïve and ART‐treated PLWH initiating PI‐based ART were 39% less likely to experience ≥5% weight gain than those initiating INSTI‐based ART [9]. Additionally, a study in virally controlled women found that metabolic changes, such as increases in haemoglobin A1c (HbA1c) and blood pressure, were greater among those initiating INSTI‐based compared to non‐INSTI‐based ART [11]. Further, a study on treatment‐naïve PLWH who received an INSTI reported a correlation between an increase in systolic blood pressure and weight gain [12].

While previous studies have evaluated the association between the use of different ART regimens and weight gain, there is limited information on the association between exposure to specific core ART agents and new‐onset cardiometabolic disease. To address this important clinical topic, the present study compared the incidence of cardiometabolic outcomes among treatment‐naïve PLWH initiated on INSTI‐based and non‐INSTI‐based ART (i.e. PI or NNRTI) using administrative claims data in the US.

## 2 METHODS

### 2.1 Data source

Data were obtained from the IBM^®^ MarketScan^®^ Commercial Claims and Encounters and Medicare Supplemental (12 August 2012−31 January 2021) and Multi‐State Medicaid claims databases (12 August 2012−30 June 2020).

These databases contain medical and pharmacy claims from approximately 100 payers (date of death and laboratory variables not available). The Commercial/Medicare Supplemental administrative claims data (recorded based on services claimed to insurance providers) come from a selection of large employers, health plans, and government and public organizations across the US, representing nearly 240 million covered individuals, including employees and their dependents, self‐insured employers and Medicare‐eligible retirees with employer‐provided Medicare Supplemental plans. The Multi‐State Medicaid Database contains seven million enrolees from 11 states and includes inpatient services, prescription drug claims, as well as information on enrolment, long‐term care and other medical care. The data are de‐identified and compliant with the Health Insurance Portability and Accountability Act and the principles of the Declaration of Helsinki.

### 2.2 Study design and exposure

A retrospective longitudinal cohort study design was used. The index date was the date of initiation of an INSTI‐ or non‐INSTI‐based (i.e. PI‐based or NNRTI‐based) ART regimen after 12 August 2013 (approval date of the first second‐generation INSTI agent in the United States, dolutegravir). For single‐tablet regimens (STRs), the index date was defined as the date of the first claim for the STR. PLWH treated with multiple‐tablet regimens (MTRs) were included if the INSTI or non‐INSTI agent was received as part of an ART regimen with ≥2 nucleoside reverse transcriptase inhibitors (NRTIs) or 1 NRTI if the regimen was dolutegravir+lamivudine, so long as all NRTI agents were received within 14 days before or after the date of the first claim for the index INSTI or non‐INSTI agent. For MTRs, the index date was defined as the date of the claim for the INSTI or non‐INSTI agent used when initiating the regimen. PLWH were assigned to either an INSTI‐based or non‐INSTI‐based cohort derived from the class of core agent in the index ART regimen. To ascertain that the index date was the date of initiation of the ART regimen of interest and that PLWH were treatment‐naïve, a 12‐month washout period without any claims for INSTI, PI or NNRTI agents pre‐index was applied. The 12‐month period of continuous insurance eligibility pre‐index was also defined as the baseline period and was used to evaluate demographic and clinical characteristics; the follow‐up period spanned from the index date until the earliest of a switch to a new ART medication outside of the index regimen class, 90 days following the discontinuation of the index medication class, end of continuous insurance eligibility or end of data availability.

### 2.3 Study population

**Figure 1:**
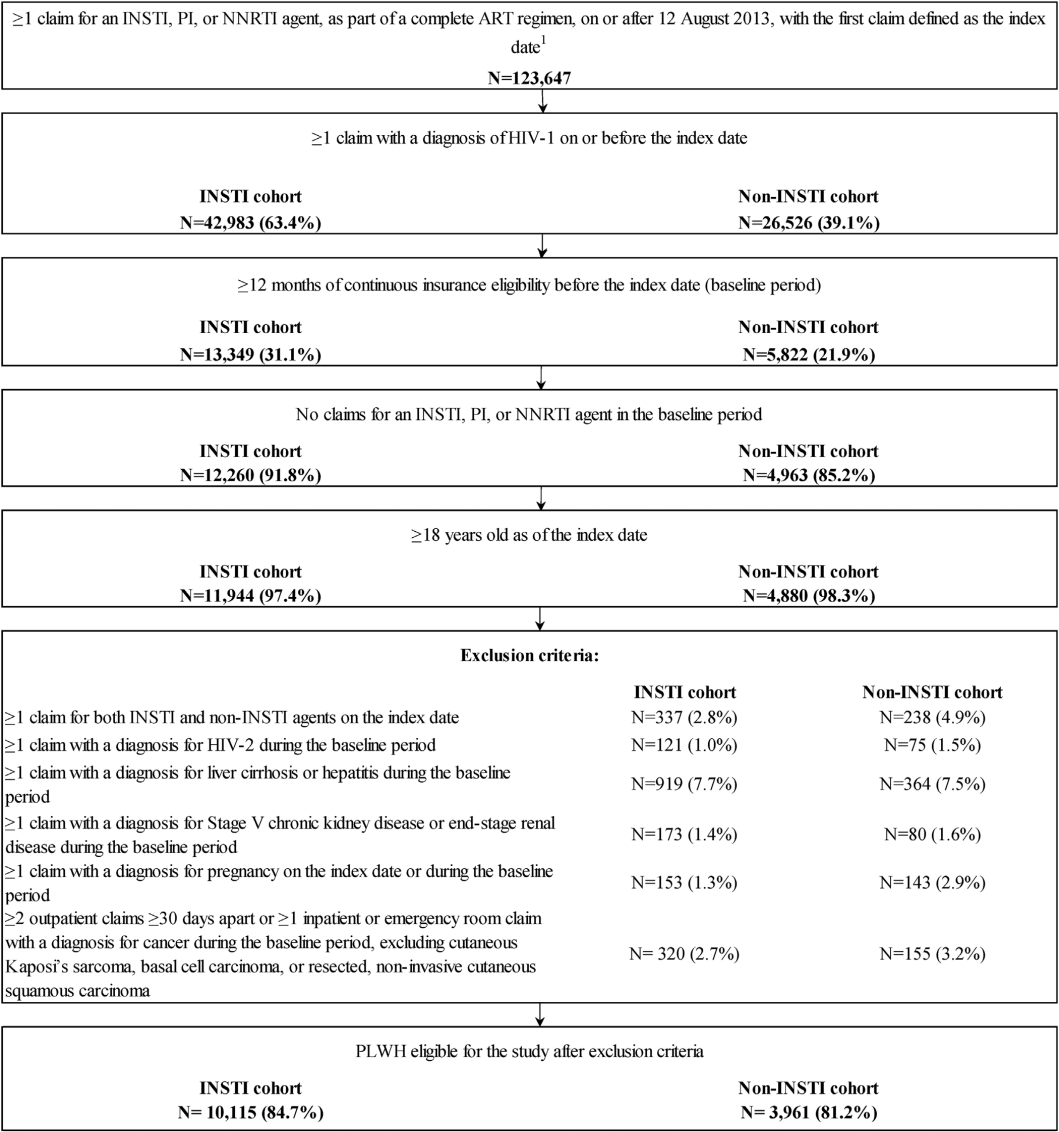
Identification of the study population. Abbreviations: ART, antiretroviral therapy; FDA, Food and Drug Administration; INSTI, integrase strand transfer inhibitor; NNRTI, non‐nucleoside reverse transcriptase inhibitor; PI, protease inhibitor; PLWH, people living with HIV‐1. Note: 1. Date of FDA approval for dolutegravir, the first approved second‐generation INSTI.

Treatment‐naïve PLWH aged ≥18 years who initiated an INSTI or non‐INSTI‐based ART regimen after 12 August 2013 were included if they had ≥1 diagnosis of HIV‐1 on or before the index date, ≥12 months of continuous insurance eligibility before the index date and no exposure to PI, INSTI or NNRTI agents during the baseline period (Figure 1).

PLWH were excluded if they initiated both an INSTI and non‐INSTI agent on the index date (since it would not allow for the exclusive classification of these individuals into either the INSTI or non‐INSTI cohort) or had ≥1 claim with a diagnosis of HIV‐2, liver cirrhosis or hepatitis, stage V chronic kidney disease or end‐stage renal disease, or pregnancy during the baseline period. PLWH were also excluded if they had ≥2 outpatient claims ≥30 days apart with a diagnosis for cancer or ≥1 inpatient or emergency room (ER) claim with a diagnosis for cancer (excluding cutaneous Kaposi's sarcoma, basal cell carcinoma or resected, non‐invasive cutaneous squamous carcinoma) during the baseline period [13, 14]. These exclusion criteria were based on prescribing information and pivotal ART trials [15, 16, 17, 18]. This resulted in the identification of 10,115 INSTI and 3961 non‐INSTI initiators.

### 2.4 Study outcomes

The following incident cardiometabolic outcomes were evaluated, separately, during the follow‐up period: congestive heart failure (CHF), coronary artery disease, myocardial infarction, stroke/transient ischemic attack (TIA), hypertension, lipid disorders (i.e. hypercholesterolaemia, hyperglyceridaemia and hyperlipidaemia), lipodystrophy, metabolic syndrome and type II diabetes mellitus (see Table S1 for the International Classification of Disease, Ninth/Tenth Revision, Clinical Modification diagnosis codes). Incident outcomes were confirmed if there were ≥2 outpatient claims ≥30 days apart or ≥1 inpatient or ER claim with a diagnosis for the conditions [13, 14]. PLWH were considered at risk of having an incident outcome if they did not have a diagnosis or medication related to the specific condition (e.g. antidiabetic medication as a proxy for type II diabetes mellitus; see Table S2 for the list of medications) during the baseline period.

In addition, the following composite outcomes were evaluated: (1) incident cardiometabolic outcome; (2) incident cardiovascular outcome, including CHF, coronary artery disease, myocardial infarction and stroke/TIA; and (3) incident metabolic outcome, including hypertension, lipid disorders, lipodystrophy, metabolic syndrome and type II diabetes mellitus. For each composite outcome, PLWH who had all conditions during the baseline period comprising the composite outcome were excluded. PLWH were considered to remain at risk for developing a condition that was part of the list of conditions included in the composite outcome if they did not have that specific condition during the baseline period.

### 2.5 Statistical analysis

Baseline characteristics were reported using means, standard deviations (SDs), medians and interquartile ranges (IQRs) for continuous variables, and counts and proportions for categorical variables. To isolate the effect of treatment on the incidence of cardiometabolic outcomes, PLWH in the INSTI and non‐INSTI cohorts were weighted using inverse probability of treatment weights (IPTW) [19, 20], which results in more statistically efficient estimates, allows an assessment of the balance for measured baseline characteristics between the treatment groups and allows any additional adjustment for residual confounding through doubly robust weighted adjustment. IPTW were constructed using the propensity score (PS), which was estimated using a logistic regression model, where the dependent variable was the index treatment (i.e. INSTI or non‐INSTI) and baseline characteristics were independent variables used to predict treatment assignment. The following baseline variables were included in the PS model: age, sex at birth, race/ethnicity, US geographic region, insurance plan type, index date calendar year, Quan−Charlson Comorbidity Index score [21] (excluding HIV‐1 symptoms), use of medications associated with weight gain and weight loss, respectively (Table S2). IPTW for each individual were constructed as follows: 1/PS for PLWH in the INSTI cohort and 1/(1‐PS) for PLWH in the non‐INSTI cohort. In addition, weights were normalized by the mean weight (i.e. each PLWH's weight was divided by the overall mean weight for the entire population; Table S3) [22]. The resulting weighted samples constituted pseudo‐populations in the INSTI‐ and non‐INSTI‐treated groups, with each individual contributing to analyses according to their reweighted representation. The resulting differences in the incidence of cardiometabolic outcomes between the weighted INSTI and non‐INSTI cohorts reflected the average treatment effect.

Differences in baseline characteristics between the two cohorts were evaluated using standardized differences (standardized difference <10% considered balanced) [23]. The incidence rate (per‐thousand person‐years; PTPY) of each cardiometabolic outcome was assessed in the weighted cohort by dividing the number of incident events observed by the total follow‐up time available multiplied by 1000. For PLWH experiencing the incident event, follow‐up was censored at the date the event first occurred. To appropriately account for the timing of the incident event and censoring, adjusted hazard ratios (HRs) obtained from multivariable weighted Cox proportional hazards models were used to compare the risk of incident cardiometabolic outcome between the INSTI and non‐INSTI cohorts. All weighted models were adjusted by covariates capturing the use of NRTIs in the index ART regimen: abacavir, emtricitabine, lamivudine, tenofovir alafenamide (TAF) and tenofovir disoproxil fumarate; 95% confidence intervals (CIs) and *p*‐values were generated using non‐parametric bootstraps with 500 replications. When estimating each treatment effect, the non‐INSTI cohort was used as the referent. As TAF has been potentially associated with weight gain [7], an exploratory, descriptive analysis was conducted among the subgroup of PLWH who used TAF as part of their index regimen. All analyses were conducted using SAS Enterprise Guide 7.1 (SAS Institute, Cary, NC).

## 3 RESULTS

Overall, 10,115 PLWH were eligible for inclusion in the INSTI cohort and 3961 in the non‐INSTI cohort (Figure 1). After applying IPTW, the weighted sample sizes were 7059 and 7017 PLWH in the INSTI and non‐INSTI cohorts, respectively (Table 1). The mean lengths of follow‐up were 1.5 years (SD = 1.5; median [IQR] = 1.0 [0.4−2.2]) in the INSTI cohort and 1.1 years (SD = 1.2; median [IQR] = 0.7 [0.3−1.4]) in the non‐INSTI cohort.

**Table 1:** Baseline characteristics during the 12‐month period prior to the index date

	Weighted population^a^		
	INSTI cohort *N* = 7059	Non‐INSTI cohort *N* = 7017	Standardized difference^b^
Age at index date (years), mean ± SD (median [IQR])	38.6 ± 12.6 (37.0 [28.0−49.0])	39.0 ± 12.6 (38.0 [28.0−49.0])	3.3%
Age categories at index date (years), *n* (%)			
18−24	1091 (15.5)	1038 (14.8)	1.8%
25−34	1939 (27.5)	1907 (27.2)	0.7%
35−44	1583 (22.4)	1593 (22.7)	0.6%
45−54	1499 (21.2)	1520 (21.7)	1.1%
55−64	875 (12.4)	890 (12.7)	0.9%
≥65	72 (1.0)	69 (1.0)	0.3%
Sex at birth, *n (%)*			
Female,	1641 (23.2)	1674 (23.9)	1.4%
Race/ethnicity, *n* (%)			
Black	1347 (19.1)	1313 (18.7)	0.9%
White	396 (5.6)	382 (5.4)	0.8%
Hispanic	60 (0.9)	66 (0.9)	1.0%
Other	48 (0.7)	45 (0.6)	0.5%
Unknown	244 (3.5)	243 (3.5)	0.1%
Unavailable^c^	4965 (70.3)	4968 (70.8)	1.0%
US geographic region^d^, *n* (%)			
South	2961 (41.9)	2986 (42.6)	1.2%
Northeast	748 (10.6)	759 (10.8)	0.7%
West	625 (8.8)	615 (8.8)	0.3%
North central	600 (8.5)	574 (8.2)	1.2%
Unknown	31 (0.4)	34 (0.5)	0.7%
Unavailable^e^	2094 (29.7)	2049 (29.2)	1.0%
Insurance plan type, *n* (%)			
Commercial only	4898 (69.4)	4904 (69.9)	1.1%
Medicaid	2094 (29.7)	2049 (29.2)	1.0%
Commercial and Medicare	48 (0.7)	44 (0.6)	0.5%
Medicare only	20 (0.3)	20 (0.3)	0.0%
Type of healthcare plan, *n* (%)			
PPO	2593 (36.7)	2618 (37.3)	1.2%
HMO	1555 (22.0)	1498 (21.4)	1.6%
Comprehensive	1258 (17.8)	1268 (18.1)	0.6%
CDHP	645 (9.1)	632 (9.0)	0.5%
POS	558 (7.9)	554 (7.9)	0.0%
HDHP	336 (4.8)	350 (5.0)	1.0%
EPO	78 (1.1)	57 (0.8)	3.0%
Unknown	35 (0.5)	40 (0.6)	1.0%
Year of index date, *n* (%)			
2013	456 (6.5)	455 (6.5)	0.1%
2014	1138 (16.1)	1140 (16.3)	0.4%
2015	952 (13.5)	955 (13.6)	0.4%
2016	1094 (15.5)	1078 (15.4)	0.4%
2017	1020 (14.5)	1028 (14.7)	0.6%
2018	910 (12.9)	907 (12.9)	0.1%
2019	780 (11.0)	770 (11.0)	0.2%
2020	671 (9.5)	649 (9.2)	0.9%
2021	39 (0.6)	34 (0.5)	1.0%
Quan−CCI (excluding HIV‐1 symptoms), mean ± SD (median [IQR])	0.5 ± 1.1 (0.0 [0.0−1.0])	0.5 ± 1.0 (0.0 [0.0−1.0])	1.0%
Medications associated with weight gain, *n* (%)^f^	2579 (36.5)	2562 (36.5)	0.0%
Medications associated with weight loss, *n* (%)^g^	631 (8.9)	631 (9.0)	0.2%

Abbreviations: CCI, Charlson Comorbidity Index; CDHP, consumer‐driven health plan; EPO, exclusive provider organization; HDHP, high‐deductible health plan; HMO, health maintenance organization; INSTI, integrase strand transfer inhibitor; IQR, interquartile range; PLWH, people living with HIV‐1; POS, point‐of‐service; PPO, preferred provider organization; SD, standard deviation.

^a^ Of note, the number of PLWH reported in this weighted population represent the sum of weights for the corresponding PLWH, rounded to the nearest integer. The proportions displayed were calculated prior to the rounding and may be slightly different than if they were calculated based on rounded numbers.

^b^ For continuous variables, the standardized difference is calculated by dividing the absolute difference in means of the INSTI cohort and non‐INSTI cohort by the pooled standard deviation of both groups. The pooled standard deviation is the square root of the average of the squared standard deviations. For categorical variables with two levels, the standardized difference is calculated using the following equation where P is the respective proportion of participants in each group: (P_INSTI_−P_non‐INSTI_)/√([p1+ p2]/2), where p1 = P_INSTI_(1−P_INSTI_) and p2 = P_non‐INSTI_(1−P_non‐INSTI_).

^c^ Race/ethnicity was only available among PLWH identified in the Multi‐State Medicaid claims database.

^d^ US geographic region was based on the US Census Bureau Regions and Divisions classification (https://www.census.gov/programs‐surveys/economic‐census/guidance‐geographies/levels.html).

^e^ US geographic region was only available among PLWH identified in the Commercial Claims and Encounters and Medicare Supplemental databases.

^f^ Medications considered were: anticonvulsants (divalproex, pregabalin, perampanel), antidepressants (escitalopram, citalopram, tricyclic antidepressants, mirtazapine, paroxetine, monoamine oxidase inhibitors), antidiabetic medications (insulins, sulfonylureas, thiazolidinediones, meglitinides), antipsychotics (quetiapine, olanzapine, risperidone, clozapine, thioridazine), corticosteroids, antihistamines (cyproheptadine), beta blockers, alpha blockers, hormonal therapy and appetite stimulants.

^g^ Medications considered were: anticonvulsants (topiramate, lamotrigine, zonisamide, felbamate, stiripentol), antidepressants (bupropion, venlafaxine, desvenlafaxine), antidiabetic medications (sodium‐glucose cotransporter‐2 inhibitors, glucagon‐like peptide‐1 agonists, pramlintide), antipsychotics (ziprasidone), growth hormone–releasing hormone (tesamorelin, ipamorelin, sermorelin), attention deficit hyperactivity disorder medications, appetite suppressants and anti‐obesity medications.

### 3.1 Study population characteristics

Prior to applying IPTW, PLWH in the INSTI cohort were younger (mean age: 37.9 years) and less likely to be female (21.7%) than those in the non‐INSTI cohort (mean age: 40.4 years; 26.6% female; Table S4). After applying IPTW, baseline characteristics were well balanced (all standardized differences <10%) between the study cohorts (Table 1). The mean ages were 38.6 years (SD = 12.6) and 39.0 years (SD = 12.6) in the INSTI and non‐INSTI cohorts, respectively; 23.2% and 23.9% of PLWH were females in the two cohorts, respectively.

During the baseline period, 39.2% of PLWH in the INSTI cohort and 40.6% in the non‐INSTI cohort had ≥1 cardiometabolic condition (Table 2). Metabolic conditions (INSTI cohort: 37.6%; non‐INSTI cohort: 39.1%) were more prevalent than cardiovascular conditions (INSTI cohort: 24.4%; non‐INSTI cohort: 25.2%) in the baseline period.

**Table 2:** Cardiometabolic conditions in the 12‐month period prior to the index date

	Weighted population^a^		
	INSTI cohort	Non‐INSTI cohort	Standardized difference^b^
	*N* = 7059	*N* = 7017	
Composite cardiometabolic conditions^c^, *n* (%)			
Any of the cardiometabolic conditions	2767 (39.2)	2849 (40.6)	2.8%
Any of the cardiovascular conditions	1722 (24.4)	1768 (25.2)	1.8%
Any of the metabolic conditions	2655 (37.6)	2741 (39.1)	3.0%
Cardiovascular conditions^c^, *n* (%)			
Coronary artery disease	1403 (19.9)	1419 (20.2)	0.9%
CHF	1206 (17.1)	1232 (17.6)	1.3%
Myocardial infarction	1108 (15.7)	1158 (16.5)	2.2%
Stroke/transient ischemic attack	264 (3.7)	279 (4.0)	1.2%
Metabolic conditions^c^, *n* (%)			
Hypertension	1977 (28.0)	2049 (29.2)	2.7%
Lipid disorders (i.e. hypercholesterolaemia, hyperglyceridaemia and hyperlipidaemia)	1331 (18.9)	1473 (21.0)	5.4%
Type II diabetes mellitus	595 (8.4)	573 (8.2)	0.9%
Metabolic syndrome	18 (0.3)	21 (0.3)	0.7%
Lipodystrophy	13 (0.2)	16 (0.2)	1.0%

Abbreviations: CHF, congestive heart failure; ICD‐9 CM/ICD‐10 CM, International Classification of Disease, Ninth/Tenth Revision, Clinical Modification; INSTI, integrase strand transfer inhibitor.

^a^ Of note, the number of PLWH reported in this weighted population represent the sum of weights for the corresponding PLWH, rounded to the nearest integer. The proportions displayed were calculated prior to the rounding and may be slightly different than if they were calculated based on rounded numbers.

^b^ For continuous variables, the standardized difference is calculated by dividing the absolute difference in means of the INSTI cohort and non‐INSTI cohort by the pooled standard deviation of both groups. The pooled standard deviation is the square root of the average of the squared standard deviations. For categorical variables with two levels, the standardized difference is calculated using the following equation where P is the respective proportion of participants in each group: (P_INSTI_−P_non‐INSTI_)/√([p1+ p2]/2), where p1 = P_INSTI_(1−P_INSTI_) and p2 = P_non‐INSTI_(1−P_non‐INSTI_).

^c^ A list of ICD‐9 CM/ICD‐10 CM codes used to identify the cardiometabolic conditions can be found in Table S1.

In the INSTI cohort, as part of the index regimen, most PLWH used elvitegravir (43.4%) or dolutegravir (33.3%; Table 3). In the non‐INSTI cohort, 60.5% of PLWH used an NNRTI as part of the index regimen (mostly rilpivirine‐based [30.4%] and efavirenz‐based [28.3%]), and the remaining 39.5% used a PI as part of the index regimen (mostly darunavir‐based [31.5%], with 7.4% specifically using darunavir/cobicistat/emtricitabine/TAF). The proportion of PLWH using TAF as part of the index regimen was 41.8% among the INSTI cohort and 32.1% among the non‐INSTI cohort.

**Table 3:** Antiretroviral medications used as part of the index regimen

	Weighted population^a^		
	INSTI cohort	Non‐INSTI cohort	Standardized difference^b^
	*N* = 7059	*N* = 7017	
ARV use, *n* (%)			
INSTI‐based	7059 (100.0)	0 (0.0)	–
Elvitegravir‐based	3061 (43.4)	0 (0.0)	–
Dolutegravir‐based	2347 (33.3)	0 (0.0)	–
BIC/FTC/TAF‐based	1299 (18.4)	0 (0.0)	–
Raltegravir‐based	352 (5.0)	0 (0.0)	–
NNRTI‐ or PI‐based	0 (0.0)	7017 (100.0)	–
NNRTI‐based	0 (0.0)	4246 (60.5)	–
Efavirenz‐based	0 (0.0)	1986 (28.3)	–
Rilpivirine‐based	0 (0.0)	2130 (30.4)	–
Doravirine‐based	0 (0.0)	39 (0.6)	–
Other NNRTI‐based	0 (0.0)	91 (1.3)	–
PI‐based	0 (0.0)	2770 (39.5)	–
Darunavir‐based	0 (0.0)	2212 (31.5)	–
Atazanavir‐based	0 (0.0)	438 (6.2)	–
Other PI‐based	0 (0.0)	121 (1.7)	–
NRTIs used as part of the index ART regimen^c^, *n* (%)			
FTC	5453 (77.2)	6670 (95.1)	53.4%
TAF	2953 (41.8)	2253 (32.1)	20.2%
TDF	2514 (35.6)	4487 (63.9)	59.1%
Lamivudine	1600 (22.7)	324 (4.6)	54.5%
Abacavir	1517 (21.5)	206 (2.9)	59.1%
Zidovudine	27 (0.4)	119 (1.7)	12.9%
Stavudine	0 (0.0)	5 (0.1)	–
Didanosine	0 (0.0)	4 (0.1)	–
Zalcitabine	0 (0.0)	0 (0.0)	–

Abbreviations: ART, antiretroviral therapy; ARV, antiretroviral; BIC, bictegravir; FTC, emtricitabine; INSTI, integrase strand transfer inhibitor; NNRTI, non‐nucleoside reverse transcriptase inhibitor; NRTI, nucleoside reverse transcriptase inhibitor; PI, protease inhibitor; PLWH, people living with HIV‐1; TAF, tenofovir alafenamide; TDF, tenofovir disoproxil fumarate.

^a^ Of note, the number of PLWH reported in this weighted population represent the sum of weights for the corresponding PLWH, rounded to the nearest integer. The proportions displayed were calculated prior to the rounding and may be slightly different than if they were calculated based on rounded numbers.

^b^ For continuous variables, the standardized difference is calculated by dividing the absolute difference in means of the INSTI cohort and non‐INSTI cohort by the pooled standard deviation of both groups. The pooled standard deviation is the square root of the average of the squared standard deviations. For categorical variables with two levels, the standardized difference is calculated using the following equation where P is the respective proportion of participants in each group: (P_INSTI_−P_non‐INSTI_)/√([p1+ p2]/2), where p1 = P_INSTI_(1−P_INSTI_) and p2 = P_non‐INSTI_(1−P_non‐INSTI_).

^c^ The sum of PLWH may be greater than the overall value since PLWH can use more than one NRTI on the index date. While imbalances remained following IPTW, the type of NRTI used was adjusted for as part of the outcome model (Cox proportional hazards model).

### 3.2 Comparison of incident cardiometabolic outcomes

**Figure 2:**
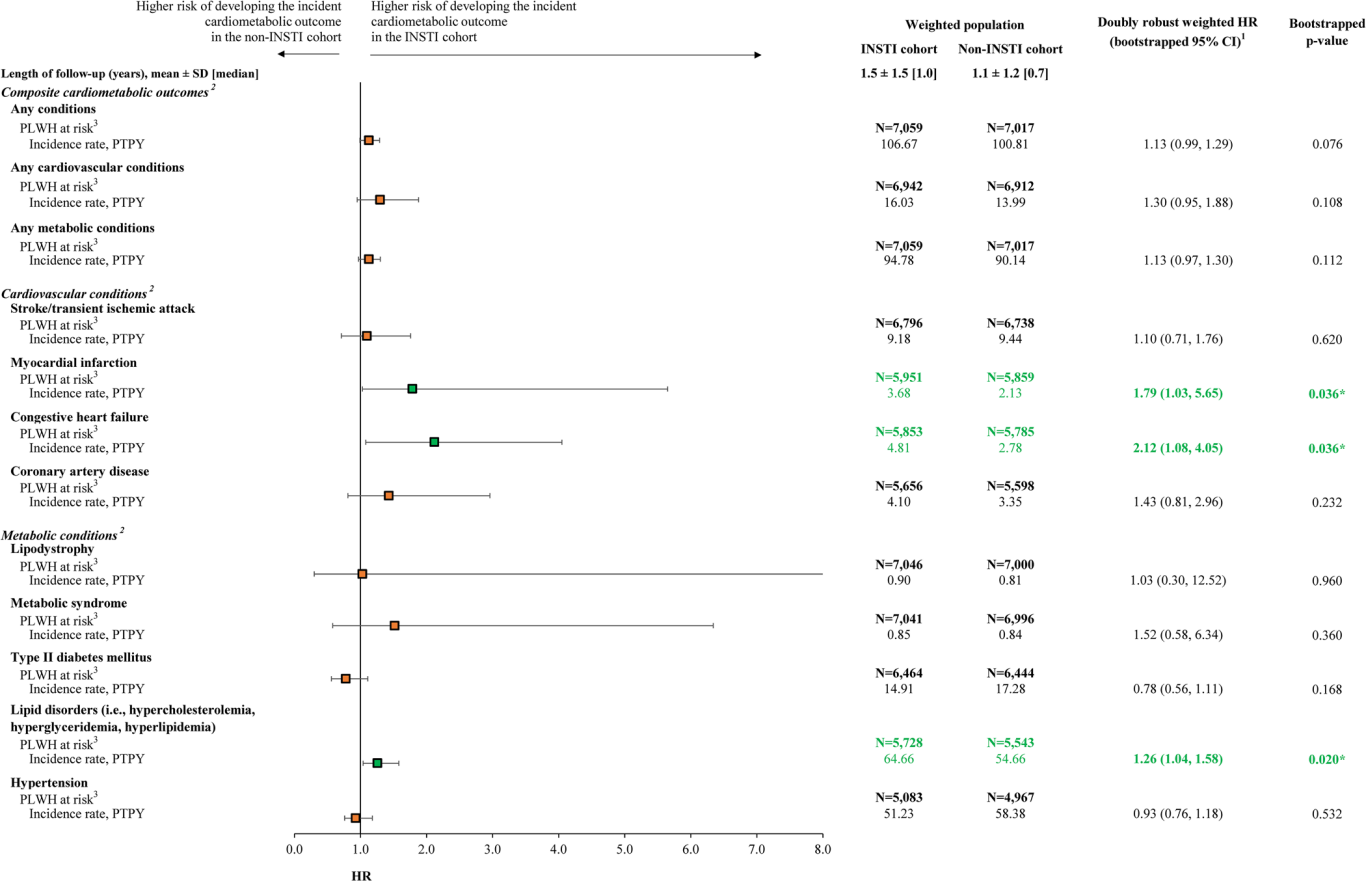
Comparison of time to incident cardiometabolic outcomes between PLWH initiated on INSTI versus non‐INSTI regimens. Abbreviations: CI, confidence interval; HR, hazards ratio; ICD‐9 CM/ICD‐10 CM, International Classification of Disease, Ninth/Tenth Revision, Clinical Modification; INSTI, integrase strand transfer inhibitor; PLWH, people living with HIV‐1; PTPY, per thousand person‐year; SD, standard deviation. Notes: * Significant at the 5% level 1. A hazard ratio >1 indicates that the INSTI cohort had a higher risk of developing the incident cardiometabolic outcome than the non‐INSTI cohort. 2. A list of ICD‐9 CM/ICD‐10 CM codes used to identify the cardiometabolic outcomes can be found in Table S1. 3. PLWH at risk represent PLWH who did not have the cardiometabolic condition of interest during the baseline period (i.e., the 12‐month period prior to the index date). A condition was considered eligible for evaluation as part of each composite outcome if the individual did not have the condition during the baseline period.

During the follow‐up period, the overall incidence rate of having any incident cardiometabolic outcome was 106.7 and 100.8 PTPY in the INSTI and non‐INSTI cohorts, respectively (Figure 2).

After adjustment, PLWH in the INSTI cohort were 2.12 times more likely to experience CHF (HR = 2.12, CI = 1.08−4.05, *p* = 0.036; incidence rate in the INSTI cohort: 4.8 PTPY; incidence rate in the non‐INSTI cohort: 2.8 PTPY), 1.79 times more likely to experience myocardial infarction (HR = 1.79, CI = 1.03−5.65, *p* = 0.036; INSTI: 3.7 PTPY; non‐INSTI: 2.1 PTPY) and 1.26 times more likely to experience lipid disorders (HR = 1.26, CI = 1.04−1.58, *p* = 0.020; INSTI: 64.7 PTPY; non‐INSTI: 54.7 PTPY) than PLWH in the non‐INSTI cohort (Figure 2).

There were no statistically significant differences observed in the likelihood of experiencing an incident cardiometabolic outcome (HR = 1.13, CI = 0.99−1.29, *p* = 0.076), an incident cardiovascular outcome (HR = 1.30, CI = 0.95−1.88, *p* = 0.108) or an incident metabolic outcome (HR = 1.13, CI = 0.97−1.30, *p* = 0.112; Figure 2) between INSTI and non‐INSTI cohorts. Further, no statistically significant differences were observed for stroke/TIA, coronary artery disease, lipodystrophy, metabolic syndrome, type II diabetes mellitus and hypertension (all *p*≥0.05; Figure 2).

Among the subgroup of PLWH using a TAF‐containing index regimen, PLWH in the INSTI cohort were approximately four times more likely to experience CHF or myocardial infarction (Table S5).

## 4 DISCUSSION

In this large retrospective longitudinal cohort study, PLWH initiated on INSTI‐based ART were at a clinically and statistically significant increased risk of developing certain cardiometabolic outcomes, including CHF, myocardial infarction and lipid disorders, compared with those initiated on non‐INSTI‐based ART. Furthermore, despite not being statistically significant, a numerically higher risk was observed for almost all outcomes in the INSTI versus non‐INSTI cohort. While the onset of cardiometabolic outcomes requires sufficiently long follow‐up to be observed (e.g. 5–10 years) in many clinical studies [24, 25, 26], significant differences in cardiometabolic risk between the INSTI and non‐INSTI cohorts were observed even in the short period of follow‐up within this study. The impact of these differences in treatment‐naïve PLWH over longer periods of observation remains unknown, but with additional follow‐up time, it is likely that observed differences in cardiometabolic risk may increase, as more PLWH experience these and related outcomes that have long latency periods.

Research on the consequence of ART use on cardiometabolic outcomes has started to emerge recently [11, 27–31], owing to growing evidence on the association of some ART with weight gain [7–9, 32–35]. In a US analysis of INSTI‐associated metabolic changes in virally controlled women enrolled in the longitudinal Women's Interagency HIV Study (WIHS), PLWH who switched to or added an INSTI to their ART regimen had significant increases in HbA1c and blood pressure relative to those who remained on a non‐INSTI ART regimen after a median follow‐up of approximately 2 years [11]. As with the current study, the WIHS analysis did not observe a difference in the incidence of diabetes mellitus or hypertension in their cohort [11]. It is, however, notable that changes in key parameters, such as HbA1c and blood pressure, have been associated with increased risks of subsequent major cardiometabolic events (e.g. metabolic syndrome, diabetes mellitus and coronary heart disease) [36, 37, 38]. Thus, longer follow‐up may be needed to assess the impact of INSTIs on certain incident cardiometabolic diseases. Shorter follow‐up periods may also be a limiting factor in several multinational studies (follow‐up time of INSTI cohorts: 1.6−2.1 years) [27, 28, 29], which observed increased risk of some cardiometabolic parameters only with specific INSTIs [28, 29] or in certain subgroups (e.g. females with cardiovascular risk factors, such as increased weight and waist circumference) [27]. More recently, a large multicentre cohort study of PLWH in Europe and Australia reported that PLWH exposed to INSTIs had a greater risk of cardiovascular disease during the first 24 months, as compared to non‐INSTI users [30]. Alternatively, in the Data Collection on Adverse Events of Anti‐HIV Drugs (D:A:D) study published in 2018, cumulative exposure to ritonavir‐boosted darunavir, a PI, was associated with increased risk of cardiovascular disease, incongruous with results for non‐INSTI regimens in this study [39]. This finding, however, was likely explained by the large proportion of treatment‐experienced PLWH, many of whom previously used efavirenz or other older ARTs, were co‐infected with hepatitis or had experienced an AIDS‐defining event, all existing risk factors for developing cardiovascular disease [40, 41, 42]. Further research is warranted to understand differences in cardiometabolic outcomes between INSTIs, PIs, and NNRTIs and between specific regimens (which were not evaluated in this study due to the smaller sample size within these subgroups). In addition, future studies with longer follow‐up, especially among at‐risk subgroups, are warranted to provide additional insight into the impact of INSTI use on cardiometabolic outcomes.

Despite the recommendation of INSTIs as first‐line treatment for most ART‐naïve PLWH, the US DHHS guidelines highlight that greater weight gain has been associated with INSTI‐based regimens compared with boosted PI‐ or NNRTI‐based regimens. The guidelines also mention that TAF, used as a backbone agent in several INSTI‐based regimens, has been associated with weight gain in treatment‐naïve PLWH [7]. Exploratory analyses on ART containing INSTI and TAF versus those on non‐INSTI and TAF found a greater risk of CHF and myocardial infarction than what was found in the overall cohort. Thus, the combined effect of INSTI and TAF on weight gain may potentially induce a greater risk of incident cardiometabolic outcomes than INSTI alone. Potential cardiometabolic risk is also recognized in the DHHS guidelines as an important research area for understanding the clinical consequence of INSTI‐associated weight gain [7]. It has been hypothesized that INSTIs may promote adipocyte hypertrophy, which leads to global fat gain, oxidative stress, mitochondrial dysfunction and insulin resistance [43]. In line with this, a recent study among PLWH from the WIHS cohort found that changes in the metabolic pathways of PLWH who experienced weight gain after switching to INSTIs reflected insulin resistance and altered mitochondrial fuel utilization, suggesting a potential mechanistic link between INSTI‐associated weight gain and metabolic disease [44].

Furthermore, obesity and elevated body mass index (BMI) are well‐established risk factors for cardiometabolic disease [45, 46], and significantly greater weight/BMI increases have been reported among PLWH treated with INSTI versus non‐INSTI regimens [9, 32, 35]. These observations may have important clinical implications for PLWH, as it has been shown that weight gain conferred an even greater risk of incident diabetes mellitus among PLWH than among HIV‐negative individuals [47]. Additionally, certain metabolic outcomes may in turn increase the risk of developing cardiometabolic conditions [45, 46]. For instance, PLWH with diabetes mellitus have been shown to be at a higher risk of developing coronary heart disease and chronic kidney disease than PLWH without diabetes mellitus [48, 49]. Future investigations are needed to delineate the potential relationships and mechanisms of INSTI‐associated weight gain and cardiometabolic outcomes.

Findings of the current study should be interpreted in light of certain limitations. As with all claims‐based analyses, our data were subject to inaccuracies or omissions in coded diagnoses, billing and other variables. In addition, antiretroviral claims do not guarantee adherence to the regimen or that the individuals used the medication as indicated. In the current study, PLWH may be censored for various reasons (i.e. switch, discontinuation and end of eligibility/data availability), which contributed to varying lengths of follow‐up among PLWH in both cohorts. However, we assumed all censoring to be non‐informative in these analyses. Meanwhile, to improve the power for detecting an effect, a class‐based analysis was conducted, and PIs and NNRTIs were grouped together into the non‐INSTI cohort, which resulted in more heterogeneity compared with the INSTI cohort. Indeed, it remains unclear if PLWH who initiate an NNRTI are at similar risk of developing cardiometabolic disease [50], or may be at lower risk [51], relative to PI users, as both have been reported in large cohort studies. Nonetheless, the imbalance in baseline characteristics was minimized by applying IPTW. In addition, since this study population included treatment‐naïve PLWH, results may not be generalizable to treatment‐experienced PLWH. It should be noted that the IPTW model may have been subject to residual confounding due to unmeasured confounders. For example, clinical variables, such as weight, BMI, waist circumference, vitals and laboratory results (e.g. HIV‐1 viral load, CD4 count; higher viral load/lower CD4 count being associated with a higher risk of cardiometabolic outcomes [52, 53]), were not available in claims data; therefore, these variables could not be accounted for in the PS model, and thus their balance cannot be ascertained. However, the strength of confounding by these unmeasured factors would depend on their association not only with the outcomes (which has been repeatedly demonstrated), but also on their association with the choice of initial ART regimen (which may be less likely). Additionally, these clinical variables could not be used to capture some of the outcomes (e.g. high blood pressure signalling the presence of hypertension). Particularly, metabolic syndrome was likely underestimated because waist circumference, a key factor for this outcome, was unavailable. Lastly, the relatively short follow‐up period may be insufficient to detect cardiometabolic outcomes that may take several years to develop, resulting in few events observed and wide 95% CIs for some outcomes.

## 5 CONCLUSIONS

HIV‐1 has been transformed into a chronic disease owing to the advent of effective and well‐tolerated combination ART. While ART has helped increase the life expectancy of PLWH, it is important that consideration be given to ART‐associated long‐term consequences, such as the increased risk of cardiometabolic diagnoses when prescribing ART. These findings showed that even over a short average follow‐up period of <2 years, INSTI use among treatment‐naïve PLWH was associated with increased risk of several cardiometabolic outcomes, such as CHF, myocardial infarction and lipid disorders, compared to non‐INSTI‐based regimens. Further research accounting for additional potential confounders and with longer follow‐up is warranted to more accurately and precisely quantify the impact of INSTI‐containing ART on long‐term cardiometabolic outcomes.

## COMPETING INTERESTS

PFR is a consultant for Janssen Scientific Affairs, LLC. BE, CR, AS, GC‐L and MH‐L are employees of Analysis Group, Inc., a consulting company that has provided paid consulting services to Janssen Scientific Affairs, LLC, which funded the development and conduct of this study and manuscript. BKB and PD are employees of Janssen Scientific Affairs, LLC and are stockholders of Johnson & Johnson.

## AUTHORS’ CONTRIBUTIONS

BE, CR, AS, GC‐L and M‐HL contributed to the study conception and design, collection and assembly of data, and data analysis and interpretation. BKB, PD and PFR contributed to the study conception and design, data analysis and interpretation. All authors reviewed and approved the final content of this manuscript.

## FUNDING

Financial support for this research was provided by Janssen Scientific Affairs, LLC. The study sponsor was involved in several aspects of the research, including the study design, the interpretation of data, the writing of the manuscript and the decision to submit the manuscript for publication.

6

## Supporting information

Supporting Information

## Data Availability

The datasets generated and analysed during the current study are not publicly available because they were used pursuant to a data use agreement. The data are available through requests made directly to IBM.
