# Supplementary material for: Incidence of cardiometabolic outcomes among people living with HIV‐1 initiated on integrase strand transfer inhibitor versus non‐integrase strand transfer inhibitor antiretroviral therapies: a retrospective analysis of insurance claims in the United States
Source: J Int AIDS Soc. 2023 Jun 12;26(6):e26123. doi: 10.1002/jia2.26123 (PMC10258864; doi:10.1002/jia2.26123)
Supplement: Supplementary file 1 — Supporting Information [file JIA2-26-e26123-s001.docx]

# Supplementary Materials

## **Supplementary Table 1**. List of diagnosis codes for cardiometabolic outcomes

| **Medical Condition** | **ICD-9-CM Diagnosis Codes** | **ICD-10-CM Diagnosis Codes** | **Proxy Medication Classes Used for Exclusion of PLWH During the Baseline Period** |
| --- | --- | --- | --- |
| ***Metabolic Conditions*** | | | |
| Hypertension | 401.1, 401.9, 642.00-642.04, 401, 402.00-405.99, 437.2, 642.10-642.24, 642.70-642.94 | I10, O10.0, O10.9, I11-I15, I67.4, O10.1, O10.2, O10.3, O10.4, O11.1, O11.2, O11.3, O11.9, O15.2, O16.1, O16.2, O16.3, O16.9 | Antihypertensives |
| Lipid disorders | 272.0 - 272.2, 272.4 | E78.0-E78.2, E78.4, E78.5 | Bile acid sequestrants, fibrate therapy, cholesterol inhibitors, statins |
| Lipodystrophy | 272.6 | E88.1 | Growth hormone–releasing hormone |
| Metabolic syndrome | 277.7 | E88.81 | - |
| Type II diabetes mellitus | 249.00-249.91, 250.00-250.93, 648.00-648.04, 775.1 | E11 | Antidiabetics |
| ***Cardiovascular Conditions*** | | | |
| CHF | 398.91, 428.0-428.9 | I09.81, I50 | ACE inhibitors, ARBs, beta blockers, diuretics, aldosterone antagonists, digoxin (lanoxin) |
| Coronary artery disease | 414.0 | I25.1, I25.7 | Aspirin, beta blockers, calcium channel blocker, ranolazine, nitroglycerin, ACE inhibitors, ARBs, cholesterol-modifying medications |
| Myocardial infarction | 410, 412 | I21-I22, I25.2 | Antiplatelets, anticoagulants, beta‑blockers, ACE inhibitors, aspirin, thrombolytics, statins |
| Stroke/transient ischemic attack | 430-438.9 | I60-I63, I65-I69, G45 | Antiplatelets  Anticoagulants |

**Abbreviations:** ACE = angiotensin-converting enzyme, ARB, angiotensin II receptor blocker, CHF = congestive heart failure; ICD-9-CM/ICD-10-CM = International Classification of Disease, Ninth/Tenth Revision, Clinical Modification; PLWH = people living with human immunodeficiency virus.

## **Supplementary Table 2**. Medications associated with weight gain and weight loss

| **Medications Associated with Weight Gain^1,2^** | **Medications Associated with Weight Loss^1,2,3,4^** |
| --- | --- |
| **Anticonvulsants** | |
| Divalproex | Topiramate |
| Pregabalin | Lamotrigine |
| Perampanel | Zonisamide |
|  | Felbamate |
|  | Stiripentol |
| **Antidepressants** | |
| Escitalopram | Bupropion |
| Citalopram | Venlafaxine |
| Tricyclic antidepressants | Desvenlafaxine |
| Mirtazapine |  |
| Paroxetine |  |
| Monoamine oxidase inhibitors |  |
| **Antidiabetic Medications** | |
| Insulins | Sodium-glucose cotransporter-2 inhibitors |
| Sulfonylureas | Glucagon-like peptide-1 agonists |
| Thiazolidinediones | Pramlintide |
| Meglitinides |  |
| **Antipsychotics** | |
| Quetiapine | Ziprasidone |
| Olanzapine |  |
| Risperidone |  |
| Clozapine |  |
| Thioridazine |  |
| **Antihistamines** | |
| Cyproheptadine |  |
| **Growth Hormone–Releasing Hormone** | |
|  | Tesamorelin |
|  | Ipamorelin |
|  | Sermorelin |
| **Others** | |
| Corticosteroids | ADHD medications |
| Beta blockers | Appetite suppressants |
| Alpha blockers | Anti-obesity medications |
| Hormonal therapy |  |
| Appetite stimulants |  |

**Abbreviations:** ADHD = attention deficit hyperactivity disorder.

**References**:

1. Categorization of certain medications by their effects on body weight. Wolters Kluwer; www.uptodate.com.

2. Common side effects of antiseizure drugs. Wolters Kluwer; www.uptodate.com.

3. Drugs available as adjuncts to diet and exercise for treatment of obesity. Wolters Kluwer; www.uptodate.com.

4. Summary of glucose-lowering interventions. Wolters Kluwer; www.uptodate.com.

## **Supplementary Table 3**. Distribution of normalized inverse probability of treatment weights

|  | **INSTI cohort** | **Non-INSTI cohort** |
| --- | --- | --- |
| **N (study population)** | 10,115 | 3,961 |
| **N (weighted population)** | 7,059 | 7,017 |
| **Weight distribution** |  |  |
| Mean | 0.70 | 1.77 |
| Minimum | 0.52 | 0.60 |
| 5th percentile | 0.54 | 0.76 |
| 25th percentile | 0.57 | 0.94 |
| 50th percentile (median) | 0.63 | 1.30 |
| 75th percentile | 0.73 | 2.17 |
| 95th percentile | 1.14 | 4.42 |
| 99th percentile | 1.46 | 6.12 |
| Maximum | 2.21 | 7.93 |
| **Abbreviations:** INSTI = integrase strand transfer inhibitor. | | |

## **Supplementary Table 4**. Unweighted baseline characteristics

|  | **Unweighted population** | | |
| --- | --- | --- | --- |
|  | **INSTI cohort** | **Non-INSTI cohort** | **Standardized difference^1^** |
|  | **N=10,115** | **N=3,961** |  |
| **Age at index date (years), mean ± SD [median (IQR)]** | 37.9 ± 12.7  [36.0 (27.0 - 48.0)] | 40.4 ± 12.4  [41.0 (30.0 - 50.0)] | 19.9% |
| **Age categories at index date (years), n (%)** |  |  |  |
| 18-24 | 1,708 (16.9) | 478 (12.1) | 13.7% |
| 25-34 | 2,927 (28.9) | 945 (23.9) | 11.5% |
| 35-44 | 2,199 (21.7) | 936 (23.6) | 4.5% |
| 45-54 | 1,974 (19.5) | 1,021 (25.8) | 15.0% |
| 55-64 | 1,210 (12.0) | 534 (13.5) | 4.6% |
| ≥65 | 97 (1.0) | 47 (1.2) | 2.2% |
| **Sex at birth** |  |  |  |
| Female, n (%) | 2,198 (21.7) | 1,054 (26.6) | 11.4% |
| **Race/Ethnicity, n (%)** |  |  |  |
| Black | 1,908 (18.9) | 808 (20.4) | 3.9% |
| White | 626 (6.2) | 163 (4.1) | 9.4% |
| Hispanic | 87 (0.9) | 32 (0.8) | 0.6% |
| Other | 73 (0.7) | 23 (0.6) | 1.8% |
| Unknown | 324 (3.2) | 173 (4.4) | 6.1% |
| Unavailable^2^ | 7,097 (70.2) | 2,762 (69.7) | 0.9% |
| **US geographic region^3^, n (%)** |  |  |  |
| South | 4,297 (42.5) | 1,585 (40.0) | 5.0% |
| Northeast | 1,030 (10.2) | 441 (11.1) | 3.1% |
| West | 878 (8.7) | 367 (9.3) | 2.0% |
| North central | 860 (8.5) | 341 (8.6) | 0.4% |
| Unknown | 32 (0.3) | 28 (0.7) | 5.5% |
| Unavailable^4^ | 3,018 (29.8) | 1,199 (30.3) | 0.9% |
| **Insurance plan type, n (%)** |  |  |  |
| Commercial only | 7,016 (69.4) | 2,714 (68.5) | 1.8% |
| Medicaid | 3,018 (29.8) | 1,199 (30.3) | 0.9% |
| Commercial and Medicare | 56 (0.6) | 34 (0.9) | 3.6% |
| Medicare only | 25 (0.2) | 14 (0.4) | 1.9% |
| **Type of healthcare plan, n (%)** |  |  |  |
| PPO | 3,671 (36.3) | 1,476 (37.3) | 2.0% |
| HMO | 2,319 (22.9) | 798 (20.1) | 6.8% |
| Comprehensive | 1,735 (17.2) | 796 (20.1) | 7.6% |
| CDHP | 950 (9.4) | 335 (8.5) | 3.3% |
| POS | 783 (7.7) | 320 (8.1) | 1.3% |
| HDHP | 501 (5.0) | 175 (4.4) | 2.5% |
| EPO | 100 (1.0) | 41 (1.0) | 0.5% |
| Unknown | 56 (0.6) | 20 (0.5) | 0.7% |
| **Year of index date, n (%)** |  |  |  |
| 2013 | 357 (3.5) | 546 (13.8) | 37.1% |
| 2014 | 1,106 (10.9) | 1,159 (29.3) | 47.0% |
| 2015 | 1,212 (12.0) | 688 (17.4) | 15.3% |
| 2016 | 1,655 (16.4) | 532 (13.4) | 8.2% |
| 2017 | 1,586 (15.7) | 448 (11.3) | 12.8% |
| 2018 | 1,549 (15.3) | 266 (6.7) | 27.7% |
| 2019 | 1,375 (13.6) | 181 (4.6) | 31.8% |
| 2020 | 1,205 (11.9) | 133 (3.4) | 32.6% |
| 2021 | 70 (0.7) | 8 (0.2) | 7.4% |
| **Quan-CCI (excluding HIV-1 symptoms), mean ± SD [median (IQR)]** | 0.5 ± 1.1 [0.0 (0.0 - 1.0)] | 0.5 ± 1.1 [0.0 (0.0 - 1.0)] | 0.9% |
| **Medications associated with weight gain, n (%)^5^** | 3,789 (37.5) | 1,351 (34.1) | 7.0% |
| **Medications associated with weight loss, n (%)^6^** | 960 (9.5) | 306 (7.7) | 6.3% |

**Abbreviations**: CCI = Charlson Comorbidity Index; CDHP = consumer-driven health plan; EPO = exclusive provider organization; HDHP = high-deductible health plan; HMO = health maintenance organization; INSTI = integrase strand transfer inhibitor; IQR = interquartile range; PLWH = people living with HIV-1; POS = point-of-service; PPO = preferred provider organization; SD = standard deviation.

**Notes**:

1. For continuous variables, the standardized difference is calculated by dividing the absolute difference in means of the INSTI cohort and non-INSTI cohort by the pooled standard deviation of both groups. The pooled standard deviation is the square root of the average of the squared standard deviations. For categorical variables with two levels, the standardized difference is calculated using the following equation where P is the respective proportion of participants in each group: (P_INSTI_-P_non-INSTI_)/√([p1+ p2]/2), where p1=P_INSTI_(1-P_INSTI_) and p2=P_non-INSTI_(1-P_non-INSTI_).

2. Race/ethnicity was only available among PLWH identified in the Multi-State Medicaid claims database.

3. US geographic region was based on the US Census Bureau Regions and Divisions classification (https://www.census.gov/programs-surveys/economic-census/guidance-geographies/levels.html).

4. US geographic region was only available among PLWH identified in the Commercial Claims and Encounters and Medicare Supplemental databases.

5. Medications considered were: anticonvulsants (divalproex, pregabalin, perampanel), antidepressants (escitalopram, citalopram, tricyclic antidepressants, mirtazapine, paroxetine, monoamine oxidase inhibitors), antidiabetic medications (insulins, sulfonylureas, thiazolidinediones, meglitinides), antipsychotics (quetiapine, olanzapine, risperidone, clozapine, thioridazine), corticosteroids, antihistamines (cyproheptadine), beta blockers, alpha blockers, hormonal therapy, and appetite stimulants.

6. Medications considered were: anticonvulsants (topiramate, lamotrigine, zonisamide, felbamate, stiripentol), antidepressants (bupropion, venlafaxine, desvenlafaxine), antidiabetic medications (sodium-glucose cotransporter-2 inhibitors, glucagon-like peptide-1 agonists, pramlintide), antipsychotics (ziprasidone), growth hormone–releasing hormone (tesamorelin, ipamorelin, sermorelin), ADHD medications, appetite suppressants, and anti-obesity medications.

## **Supplementary Table 5**. Incident rates of cardiometabolic outcomes between PLWH initiated on INSTI with TAF vs. non-INSTI with TAF regimens

|  | **Weighted population** | | **Unadjusted weighted IRR^1^** |
| --- | --- | --- | --- |
|  | **INSTI cohort** | **Non-INSTI cohort** |  |
| **Length of follow-up (years), mean ± SD [median]** | 1.2 ± 1.1 [0.9] | 0.9 ± 0.9 [0.6] |  |
| ***Composite cardiometabolic outcomes^2^*** |  |  |  |
| **Any conditions** |  |  |  |
| PLWH at risk^3^ | **N=2,953** | **N=2,253** |  |
| Incidence rate, PTPY | 108.00 | 113.69 | 0.95 |
|  |  |  |  |
| **Any cardiovascular conditions** |  |  |  |
| PLWH at risk^3^ | **N=2,902** | **N=2,223** |  |
| Incidence rate, PTPY | 16.47 | 11.60 | 1.42 |
|  |  |  |  |
| **Any metabolic conditions** |  |  |  |
| PLWH at risk^3^ | **N=2,953** | **N=2,253** |  |
| Incidence rate, PTPY | 94.72 | 104.55 | 0.91 |
|  |  |  |  |
| ***Cardiovascular conditions^2^*** |  |  |  |
| **Stroke/transient ischemic attack** |  |  |  |
| PLWH at risk^3^ | **N=2,843** | **N=2,174** |  |
| Incidence rate, PTPY | 9.38 | 10.09 | 0.93 |
|  |  |  |  |
| **Myocardial infarction** |  |  |  |
| PLWH at risk^3^ | **N=2,498** | **N=1,896** |  |
| Incidence rate, PTPY | 3.62 | 1.08 | 3.36 |
|  |  |  |  |
| **Congestive heart failure** |  |  |  |
| PLWH at risk^3^ | **N=2,465** | **N=1,863** |  |
| Incidence rate, PTPY | 4.05 | 0.88 | 4.58 |
|  |  |  |  |
| **Coronary artery disease** |  |  |  |
| PLWH at risk^3^ | **N=2,378** | **N=1,801** |  |
| Incidence rate, PTPY | 4.56 | 0.00 | -- |
|  |  |  |  |
| ***Metabolic conditions^2^*** |  |  |  |
| **Lipodystrophy** |  |  |  |
| PLWH at risk^3^ | **N=2,950** | **N=2,250** |  |
| Incidence rate, PTPY | 0.39 | 0.94 | 0.42 |
|  |  |  |  |
| **Metabolic syndrome** |  |  |  |
| PLWH at risk^3^ | **N=2,947** | **N=2,239** |  |
| Incidence rate, PTPY | 0.55 | 0.00 | -- |
|  |  |  |  |
| **Type II diabetes mellitus** |  |  |  |
| PLWH at risk^3^ | **N=2,690** | **N=2,066** |  |
| Incidence rate, PTPY | 16.27 | 26.53 | 0.61 |
|  |  |  |  |
| **Lipid disorders (i.e., hypercholesterolemia, hyperglyceridemia, hyperlipidemia)** |  |  |  |
| PLWH at risk^3^ | **N=2,401** | **N=1,823** |  |
| Incidence rate, PTPY | 59.60 | 62.42 | 0.95 |
|  |  |  |  |
| **Hypertension** |  |  |  |
| PLWH at risk^3^ | **N=2,139** | **N=1,655** |  |
| Incidence rate, PTPY | 54.29 | 59.37 | 0.91 |

**Abbreviations:** ICD-9 CM/ICD-10 CM = International Classification of Disease, Ninth/Tenth Revision, Clinical Modification; INSTI = integrase strand transfer inhibitor; IRR = incidence rate ratio; PLWH = people living with HIV-1; PTPY = per person per-year; SD = standard deviation; TAF = tenofovir alafenamide.

**Notes**:

1. An incident rate ratio >1 indicates that the INSTI cohort had a higher incidence rate than the non-INSTI cohort.

2. A list of ICD-9 CM/ICD-10 CM codes used to identify the cardiometabolic outcomes can be found in **Supplementary Table 1**.

3. PLWH at risk represent PLWH who did not have the cardiometabolic condition of interest during the baseline period (i.e., the 12-month period prior to the index date). A condition was considered eligible for evaluation as part of each composite outcome if the individual did not have the condition during the baseline period.
